# Association between Traffic-Related Air Pollution in Schools and Cognitive Development in Primary School Children: A Prospective Cohort Study

**DOI:** 10.1371/journal.pmed.1001792

**Published:** 2015-03-03

**Authors:** Jordi Sunyer, Mikel Esnaola, Mar Alvarez-Pedrerol, Joan Forns, Ioar Rivas, Mònica López-Vicente, Elisabet Suades-González, Maria Foraster, Raquel Garcia-Esteban, Xavier Basagaña, Mar Viana, Marta Cirach, Teresa Moreno, Andrés Alastuey, Núria Sebastian-Galles, Mark Nieuwenhuijsen, Xavier Querol

**Affiliations:** 1 Centre for Research in Environmental Epidemiology (CREAL), Barcelona, Catalonia, Spain; 2 Pompeu Fabra University, Barcelona, Catalonia, Spain; 3 Consortium for Biomedical Research in Epidemiology and Public Health (CIBERESP), Madrid, Spain; 4 Institut Hospital del Mar d’Investigacions Mèdiques–Parc de Salut Mar, Barcelona, Catalonia, Spain; 5 Institute of Environmental Assessment and Water Research (IDAEA-CSIC), Barcelona, Catalonia, Spain; 6 Learning Disabilities Unit (UTAE), Neuropediatrics Department, Hospital Sant Joan de Déu, Universitat de Barcelona, Barcelona, Spain; Simon Fraser University, CANADA

## Abstract

**Background:**

Air pollution is a suspected developmental neurotoxicant. Many schools are located in close proximity to busy roads, and traffic air pollution peaks when children are at school. We aimed to assess whether exposure of children in primary school to traffic-related air pollutants is associated with impaired cognitive development.

**Methods and Findings:**

We conducted a prospective study of children (*n* = 2,715, aged 7 to 10 y) from 39 schools in Barcelona (Catalonia, Spain) exposed to high and low traffic-related air pollution, paired by school socioeconomic index; children were tested four times (i.e., to assess the 12-mo developmental trajectories) via computerized tests (*n* = 10,112). Chronic traffic air pollution (elemental carbon [EC], nitrogen dioxide [NO2], and ultrafine particle number [UFP; 10–700 nm]) was measured twice during 1-wk campaigns both in the courtyard (outdoor) and inside the classroom (indoor) simultaneously in each school pair. Cognitive development was assessed with the *n*-back and the attentional network tests, in particular, working memory (two-back detectability), superior working memory (three-back detectability), and inattentiveness (hit reaction time standard error). Linear mixed effects models were adjusted for age, sex, maternal education, socioeconomic status, and air pollution exposure at home.

Children from highly polluted schools had a smaller growth in cognitive development than children from the paired lowly polluted schools, both in crude and adjusted models (e.g., 7.4% [95% CI 5.6%–8.8%] versus 11.5% [95% CI 8.9%–12.5%] improvement in working memory, *p* = 0.0024). Cogently, children attending schools with higher levels of EC, NO2, and UFP both indoors and outdoors experienced substantially smaller growth in all the cognitive measurements; for example, a change from the first to the fourth quartile in indoor EC reduced the gain in working memory by 13.0% (95% CI 4.2%–23.1%). Residual confounding for social class could not be discarded completely; however, the associations remained in stratified analyses (e.g., for type of school or high-/low-polluted area) and after additional adjustments (e.g., for commuting, educational quality, or smoking at home), contradicting a potential residual confounding explanation.

**Conclusions:**

Children attending schools with higher traffic-related air pollution had a smaller improvement in cognitive development.

## Abstract 2

In a prospective cohort study, Jordi Sunyer and colleagues examine the association between traffic-related air pollution and 12 month cognitive development trajectories in primary school children in Barcelona.

## Editors' Summary

**Background:**

Human brain development is a complex and lengthy process. During pregnancy, the basic structures of the brain are formed, and the neural circuits that will eventually control movement, speech, memory, and other cognitive (thinking) functions, as well as the function of many organs, begin to be established. By the time of birth, the brain is about a quarter of its adult size, and the neural circuits that control vital bodily functions such as breathing are well developed. By contrast, the cerebral cortex—the brain region that is involved in thought and action—is poorly developed. Much of the development of the cerebral cortex happens during the first two years of life. For example, babies usually learn to crawl at about nine months. Other aspects of brain function take longer to develop. Thus, the cognitive functions that are essential for learning undergo considerable development between the ages of 6 and 10 years, and further brain changes occur during adolescence.

**Why Was This Study Done?:**

Exposure to the air pollutants produced by the combustion of fossil fuels by vehicles during pregnancy or infancy has been associated with delays in cognitive development. Moreover, experiments in animals suggest that traffic-related air pollution is a developmental neurotoxicant—a factor that disrupts brain development. However, although many schools are located next to busy roads and although traffic-related air pollution levels peak during school hours, it is not known whether exposure of school-age children to traffic-related air pollutants impairs their cognitive development and thus their ability to learn. Here, in a prospective cohort study (the BREATHE study), the researchers assess whether exposure of children aged 7–10 years to traffic-related air pollutants in schools in Barcelona, Spain, is associated with impaired cognitive development. A prospective cohort study is an observational investigation that studies groups (cohorts) of individuals who differ with respect to a specific factor to determine how exposure to this factor affects specific outcomes.

**What Did the Researchers Do and Find?:**

The researchers used computerized tests to measure the development of working memory (the system that holds multiple pieces of transitory information in the mind where they can be manipulated), superior working memory (working memory that involves continuous updating of the working memory buffer), and attentiveness every three months over a 12-month period in 2,715 primary school children attending 39 schools exposed to high or low levels of traffic-related air pollution and paired by socioeconomic index. That is, the researchers compared three cognitive development outcomes in the children attending each school where exposure to air pollution was high with the same outcomes in children attending a school with a similar socioeconomic index where exposure to pollution was low; school pairing was undertaken to avoid “confounding” by social class, a factor that is known to affect cognitive development. Statistical analyses of these data indicated that the increase in cognitive development over time among children attending highly polluted schools was less than that among children attending paired lowly polluted schools, even after adjusting for additional factors that affect cognitive development. Thus, for example, there was an 11.5% 12-month increase in working memory at the lowly polluted schools but only a 7.4% 12-month increase in working memory at the highly polluted schools. Other analyses indicated that children attending schools with higher levels of traffic-related air pollutants in either the courtyard or in the classroom experienced a substantially smaller increase over the 12-month study in all three cognitive measurements than those attending schools with lower levels of pollutants.

**What Do These Findings Mean?:**

These findings suggest that, compared with attendance at schools exposed to low levels of traffic-related air pollution, attendance at schools exposed to high levels of traffic-related air pollution is associated with a smaller increase in cognitive development over a 12-month period among 7- to 10-year-old children in Barcelona. The accuracy of these findings may be limited by residual confounding. That is, the children attending schools where traffic-related pollution is high might have shared other unknown characteristics that affected their cognitive development. Importantly, these findings do not prove that traffic-related air pollution causes impairment of cognitive development. Rather, they suggest that the developing brain may be vulnerable to traffic-related air pollution well into middle childhood, a conclusion that has implications for the design of air pollution regulations and for the location of new schools.

**Additional Information:**

Please access these websites via the online version of this summary at http://dx.doi.org/10.1371/journal.pmed.1001792.

- The US Centers for Disease Control and Prevention provides information about child development, including information about middle childhood (in English and Spanish)
- Tox Town is an interactive site that provides information about toxic chemicals and environmental health risks, including air pollution
- The US Environmental Protection Agency provides information about air pollution near roadways and health
- Wikipedia has pages on cognitive development and on air pollution (note that Wikipedia is a free online encyclopedia that anyone can edit; available in several languages)
- MedlinePlus provides links to additional resources about air pollution (in English and Spanish)
- The Centre for Research in Environmental Epidemiology website provides more information about the BREATHE study

## Introduction

Air pollution is a suspected developmental neurotoxicant [1]. In animals, inhalation of diesel exhaust and ultrafine particles results in elevated cytokine expression and oxidative stress in the brain [2,3] and altered animal behavior [4,5]. In children, exposure to traffic-related air pollutants during pregnancy or infancy, when the brain neocortex rapidly develops, has been related to cognitive delays [6–8].

Children spend a large proportion of their day at school, including the period when daily traffic pollution peaks. Many schools are located in close proximity to busy roads, which increases the level of traffic-related air pollution in schools and impairs children’s respiratory health [9]. There is currently very little evidence on the role of traffic-related pollution in schools on cognitive function [10]. Though the brain develops steadily during prenatal and early postnatal periods, resulting in the most vulnerable window [1], high cognitive executive functions essential for learning [11] develop significantly from 6 to 10 y of age [12]. The brain regions related to executive functions such as working memory and attention—largely the prefrontal cortex and the striatum [13]—have shown inflammatory responses after traffic-related air pollution exposure [2,14]. We aimed to assess the relationship between long-term exposure to traffic-related air pollutants at school and cognitive development measurements in primary school children within the BREATHE (Brain Development and Air Pollution Ultrafine Particles in School Children) project.

## Methods

### Funding

The research leading to these results received funding from the European Research Council under ERC Grant Agreement number 268479 for the BREATHE project.

### Design

**Fig 1:**
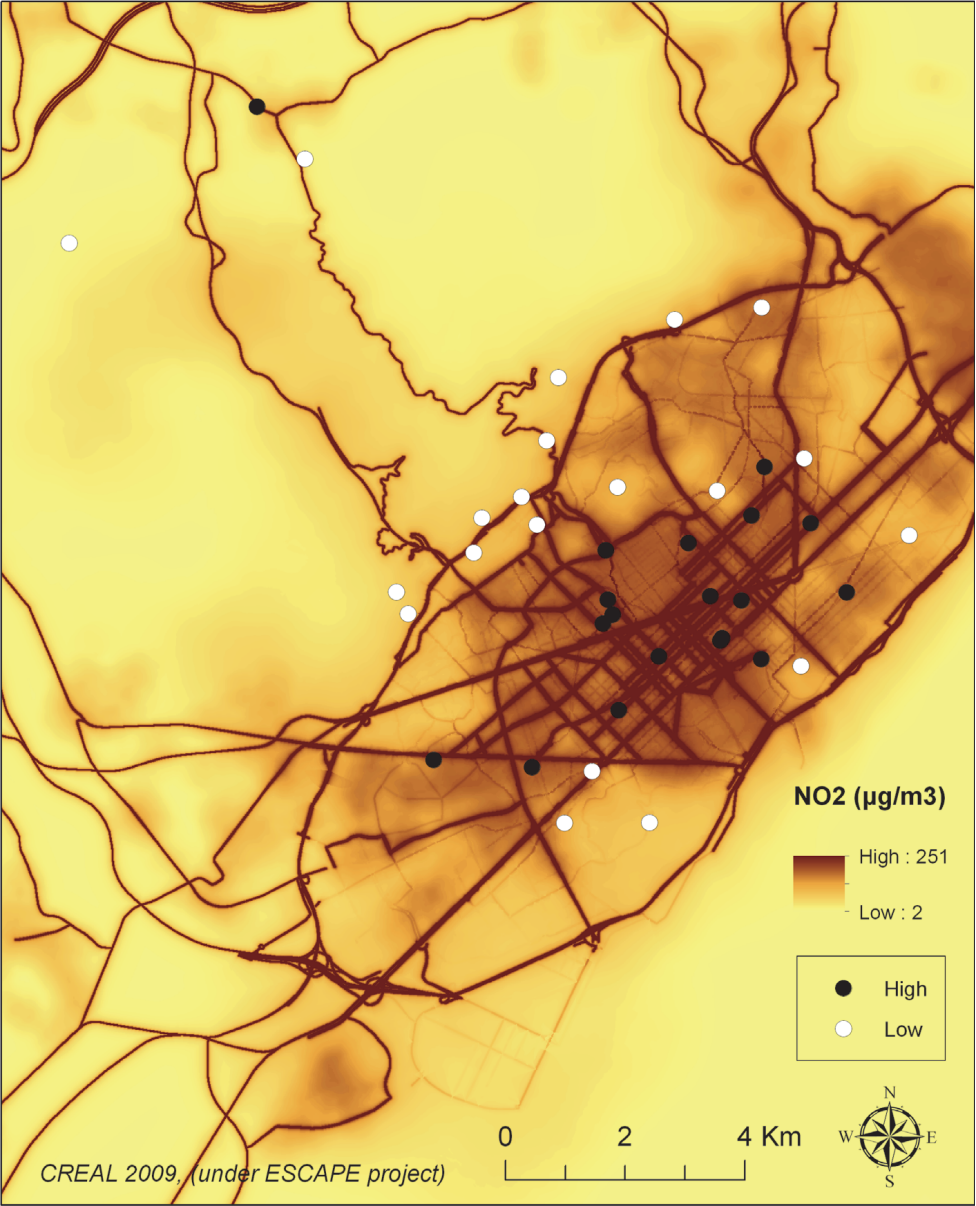
Map of Barcelona and the schools by high or low air pollution by design. Black dots indicate the locations of schools with high air pollution, and white dots indicate the locations of schools with low air pollution, based on NO_2_ levels.

Forty schools in Barcelona (Catalonia, Spain) were selected based on modeled traffic-related nitrogen dioxide (NO_2_) values [15]. Low- and high-NO_2_ schools were paired by socioeconomic vulnerability index and type of school (i.e., public/private). A total of 39 schools agreed to participate and were included in the study (Fig. 1). Participating schools were similar to the remaining schools in Barcelona in terms of socioeconomic vulnerability index (0.46 versus 0.50, Kruskal-Wallis test, *p* = 0.57) and NO_2_ levels (51.5 versus 50.9 μg/m^3^, *p* = 0.72).

All school children (*n* = 5,019) without special needs in grades 2 through 4 (7–10 y of age) were invited to participate, and families of 2,897 (59%) children agreed. All children had been in the school for more than 6 mo (and 98% more than 1 y) before the beginning of the study. All parents or guardians signed the informed consent form approved by the Clinical Research Ethical Committee (No. 2010/41221/I) of the Institut Hospital del Mar d’Investigacions Mèdiques–Parc de Salut Mar, Barcelona, Spain.

### Outcomes: Cognitive Development

Cognitive development was assessed through long-term change in working memory and attention. From January 2012 to March 2013, children were evaluated every 3 mo over four repeated visits, using computerized tests in series lasting approximately 40 min in length. We selected working memory and attention functions because they grow steadily during preadolescence [12,16]. The computerized tests chosen (the *n*-back task on working memory [12] and the attentional network test [ANT] [17]) have been validated with brain imaging [13,17] and in the general population [18]. Groups of 10–20 children were assessed together, wearing ear protectors, and were supervised by one trained examiner per 3–4 children. For the *n*-back test, we examined different *n*-back loads (up to three back) and stimuli (colors, numbers, letters, and words). For analysis here, we selected two-back and three-back loads for number and word stimuli as they showed a clear age-dependent slope in the four measurements and had little learning effect. Numbers and words activate different brain areas. The two-back test predicts general mental abilities (hereafter called working memory), while the three-back test also predicts superior functions such as fluid intelligence (hereafter called superior working memory) [19]. All sets of *n*-back tests started with colors as a training phase to ensure the participant’s understanding. The *n*-back parameter analyzed was *d* prime (*d′*), a measure of detection subtracting the normalized false alarm rate from the hit rate: (*Z*_hit rate_ − *Z*_false alarm rate_) × 100. A higher *d′* indicates more accurate test performance. Among the ANT measures, we chose hit reaction time standard error (HRT-SE) (standard error of reaction time for correct responses)—a measure of response speed consistency throughout the test [20]—since it showed very little learning effect and the clearest growth during the 1-y study period among all the ANT measurements. A higher HRT-SE indicates highly variable reactions related to inattentiveness.

### Exposures: Direct Measurements of Traffic-Related School Air Pollution

Each pair of schools was measured simultaneously twice during 1-wk periods separated by 6 mo, in the warm and cold periods of the year 2012. Indoor air in a single classroom and outdoor air in the courtyard were measured simultaneously. The pollutants measured during class time in schools were real-time concentrations of black carbon (BC) and ultrafine particle number (UFP; 10–700 nm in this study) concentration, measured using the MicroAeth AE51 (AethLabs) and DiSCmini (Matter Aerosol) meters, respectively, and 8-h (09:00 to 17:00 h) particulate matter < 2.5 μm (PM_2.5_) measured using a high-volume sampler (MCV). Details of PM_2.5_ filter chemical analysis are described elsewhere [21]. Given the high correlation between continuous BC and elemental carbon (EC) in PM_2.5_ filters (*r* = 0.95), only EC was considered here. Weekday NO_2_ was measured with one passive tube (Gradko). We selected EC, NO_2_, and UFP given their relation to road traffic emissions in Barcelona, particularly EC [21,22]. In contrast, school’s PM_2.5_ was poorly related to traffic because of the relevance of specific school sources in our study [21,23] and was not included here.

Outdoor and indoor long-term school air pollution levels were obtained by averaging the two 1-wk measures. To achieve a better spatial long-term average, EC and NO_2_ were also adjusted for temporal variability. Seasonalized levels were obtained by multiplying the daily concentration at each school by the ratio of annual average to the same day concentration at a fixed air quality background monitoring station in Barcelona, operationed continuously throughout the year, as detailed elsewhere [23]. Seasonalized measures had a stronger correlation between the first and the second campaign than non-seasonalized measures (e.g., *r* = 0.73 versus 0.61 for indoor EC and *r* = 0.64 versus 0.62 for indoor NO_2_). In contrast, seasonalized UFP had a poorer correlation between the two measurement campaigns than non-seasonalized UFP (*r* = 0.38 versus 0.70 for outdoor UFP and *r* = 0.17 versus 0.40 for indoor levels). Therefore, non-seasonalized UFP was selected in this study. The correlations between the temporally adjusted annual concentrations of EC and NO_2_ at each school and the land use regression annual estimate of BC at each school were 0.73 and 0.74, respectively, indicating good capture of the long-term average concentrations at these schools.

### Contextual and Individual Covariates

Socio-demographic factors were measured using a neighborhood socioeconomic vulnerability index (based on level of education, unemployment, and occupation in each census tract, the finest spatial census unit, with median area of 0.08 km^2^) [24] according to both the school and home address, as well as through parents’ responses to the BREATHE questionnaire on family origin, gestational age and weight, breastfeeding, parental education, occupation, marital status, smoking during pregnancy, environmental tobacco smoke at home, commuting mode, and use of computer games. Standard measurements of height and weight were performed to define overweight and obesity [25]. Attention deficit hyperactivity disorder (ADHD) symptoms (ADHD/DSM-IV Scales, American Psychiatric Association 2002) were reported by teachers. Parents completed the Strengths and Difficulties Questionnaire (SDQ) on child behavioral problems [26].

Noise in the classroom before children arrived to school (hereafter called noise) was measured as the best marker of traffic noise exposure and was included here as a covariate. Data were obtained from comprehensive noise measurements conducted during the second 1-wk campaign of air pollution sampling. Three consecutive 10-min measurements of equivalent sound pressure levels (in A-weighted decibels) at different distributed locations within the classroom were performed over two consecutive days using a calibrated SC-160 sound level meter (CESVA; ±1.0 dB tolerance [type 2], range: 30–137 dB). As we aimed to register traffic and background noise levels, any unusual sounds were deleted, and measurements were conducted before children arrived to school (before 9:00 a.m.). For robustness, we averaged the 30-min measurements from the two consecutive days, though they showed high reproducibility. Short-term noise measurements as short as 5 min have been shown to represent long-term averages [27].

Exposure at home to NO_2_ and BC (PM_2.5_ absorbance) at the time of the study was estimated at the geocoded postal address of each participant using land use regression models, details of which are explained elsewhere [15]. Similarly, school and residential surrounding greenness was measured in buffers of 100 m around the address based on the Normalized Difference Vegetation Index (NDVI) derived from Landsat 5 Thematic Mapper data. Residential history was reported by parents. The longest held address was used in 174 children (5.9%) who lived in two homes over the study period. Distance from home to school was estimated based at the geocoded postal address of each participant and school.

### Statistical Analysis

A total of 2,715 (93.7%) children with complete data (i.e., repeated outcome at least twice and individual data on maternal education and age) were included. They performed 10,112 (93.1%) tests. Because of the multilevel nature of the data (i.e., visits within children within schools), we used linear mixed effects models with the cognitive parameters (test performance) from the four repeated visits as outcomes and random effects for child and school. Age (centerd at visit 1) was included in the model in order to capture the growth trajectory of cognitive test performance. An interaction between age at each visit and school air pollution was included to capture changes in growth trajectory associated with school air pollution exposure. The main effect of air pollution (AP), which was also included in the model, captures the baseline (visit 1) differences in cognitive function that are associated with air pollution (model 1):

$$Y_{sit}=\beta_{0}+\beta_{1}({\text{Age}}_{t}-{\text{Age}}_{1})\;+\;\beta_{2}\text{AP}\;+\;\beta_{3}({\text{A ge}}_{t}-{\text{Age}}_{1})\text{AP}\;+u_{s}+v_{i(s)}+\varepsilon_{sit}$$ (1)

where *Y*_*sit*_ is the cognitive test result for subject *i* in school *s* at visit *t*, *t* = {1,2,3,4}; *u*_*s*_ is random effects at school level, assumed to be normally distributed with mean 0 and variance $\sigma_{u}^{2}$; *v*_*i*(*s*)_ is random effects associated with subject *i* in school *s*, assumed to be normally distributed with mean 0 and variance $\sigma_{v}^{2}$; and ε_sit_ is the model residuals, assumed to be normally distributed with mean 0 and variance $\sigma_{\varepsilon }^{2}$.

This model was further adjusted for potential confounders selected with directed acyclic graphs. Based on all socio-demographic and contextual covariables mentioned above, we used the program DAGitty 2.0 [28], with a priori definition of the temporal direction of the events, to draw causal diagrams. The final adjusted model (model 2) included additional coefficients for sex, maternal education (less than/primary/secondary/university), residential neighborhood socioeconomic status, and air pollution exposure at home:

$$\begin{array}{l} Y_{sit}\;=\;\beta_{0}\;+\;\beta_{1}({\text{Age}}_{\text{t}}-{\text{Age}}_{1})\;+\;\beta_{2}\text{AP}\;+\;\beta_{3}({\text{Age}}_{\text{t}}-{\text{Age}}_{1})\text{AP}\;+\;\beta_{4}\text{Sex}\;+\;\beta_{5}\text{Mat\_educ\_primary}\;+\\ \beta_{6}\text{Mat\_educ\_secondary}\;+\;\beta_{7}\text{Mat\_educ\_university}\;+\;\beta_{8}\text{Neighborhood\_socioeconomic\_status}\;+\\ \beta_{9}\text{Air\_pollution\_exposure\_at\_home}\;+u_{s}+v_{i(s)}\;+\;\varepsilon_{sit} \end{array}$$ (2)

The interactions between age and maternal education and socioeconomic status were unrelated to cognitive development (*p* = 0.33) and were not included in the models. Other variables such as quality of the test (i.e., room density and noise) and hour, day of the week, temperature, and humidity at test performance were not included in the final model after assessing their inclusion in the multivariate model and obtaining no change in the school air pollution coefficient (i.e., <1%).

School air pollution exposure was first treated as a dichotomous variable based on the high/low air pollution classification of schools used in the design stage. In a second step, we fitted the same models but replaced the binary air pollution variable by the direct measurements of air pollution levels either inside or outside the schools as quantitative exposures. Linearity of the relation between air pollution and cognitive tests was assumed since using multiple polynomial models did not improve model fit. Furthermore, to assess whether a part of our observed associations was due to potential residual confounding, models were adjusted for all covariates referred to above, both individual (e.g., smoking at home or commuting [distance and walking mode]) and contextual (e.g., greenness or noise). Sensitivity analyses were also conducted to assess effect modification by high-/low-air-pollution school, type of school, and residential neighborhood socioeconomic status in order to explore the potential for residual confounding, and by sex, maternal education, ADHD symptoms, and obesity in order to assess susceptibility. Both stratified analyses and modeling of the third-order interaction term with age, air pollution, and the third variable in the regression models were conducted.

Sample size was calculated based on a previous study that showed differences in executive function (mean 100, standard deviation 15) of four points by carbon particle interquartile range [6]. One would need 800 individuals to detect a difference of four points between the first and last categories of air pollution exposure (assuming exposure is divided into four groups according to quartiles) with a statistical power of 80% and alpha = 0.05. We tripled the number of individuals to be able to detect associations within three strata (*n* = 2,400). Analyses were conducted using R (3.0.2; R Foundation for Statistical Computing) and replicated with Stata 12 (StataCorp). Statistical significance was set at *p <* 0.05.

## Results

Children were on average 8.5 y old at baseline, and 50% were girls. The cognitive parameters improved during the 1-y follow-up period (Table 1). On average, working memory increased by 19.0%, superior working memory increased by 15.2%, and inattentiveness decreased by 19.2% (all *p* < 0.001 for linear trend). The magnitude of the 12-mo change was similar in boys and girls, with the exception of superior working memory (numbers), with a lower growth in girls (*p* = 0.001). The cognitive parameters at baseline were negatively associated with maternal education, but not their yearly change (Table 2).

**Table 1:** Description of the cognitive outcomes in children.

Visit	*n*	Age (Mean)	Working Memory (Two-Back Numbers, *d′*)	Superior Working Memory (Three-Back Numbers, *d′*)	Inattentiveness (HRT-SE, Milliseconds)
1	2,511	8.5 y	221 (131, 363)	112 (59, 188)	267 (202, 336)
2	2,593	8.7 y	222 (131, 392)	123 (59, 190)	248 (184, 318)
3	2,518	9.1 y	236 (131, 392)	129 (59, 190)	243 (181, 314)
4	2,447	9.4 y	263 (153, 392)	129 (64, 212)	224 (163, 291)

Data are median (25th, 75th percentiles).

**Table 2:** Cognitive outcomes by maternal education.

Cognitive Outcome	Non-University (*n* = 1,125)	University (*n* = 1,590)	*p*-Value^‡^
**Working memory (two-back numbers, *d′*)**			
Baseline	207 (128)	239 (122)	<0.001
12-mo change	30 (161)	29 (153)	0.759
**Superior working memory (three-back numbers, *d′*)**			
Baseline	108 (100)	127 (100)	<0.001
12-mo change	18 (132)	20 (130)	0.746
**Inattentiveness (HRT-SE, milliseconds)**			
Baseline	283 (92)	263 (88)	<0.001
12-mo change	−34 (93)	−41 (86)	0.055

Data are mean (standard deviation).

^‡^Kruskal-Wallis test.

Traffic-related air pollution levels were highly variable between schools (Table 3). EC levels were similar outdoors and indoors, while outdoor levels of NO_2_ and UFP were higher than indoor levels. EC showed a high penetration into the classrooms (indoor/outdoor ratio 94.1% [95% CI 85.7%–102.4%]), which was lower for NO_2_ (64.5% [95% CI 59.3%–69.7%]) and UFP (70.4% [95% CI 63.5%–77.3%]). Outdoor NO_2_ levels at schools were higher than urban background levels. Both during the warm and cold seasons, EC and NO_2_ had strong indoor–outdoor correlations, while the correlation was moderate for UFP (Table 4). EC had a strong correlation with NO_2_ and with UFP during the warm and cold seasons both outdoors and indoors. EC indoors and UFP outdoors showed the highest correlation between the two seasons. In relation to the covariates, EC and NO_2_ were not correlated with the socioeconomic vulnerability index of the school (*r* = 0.10 and 0.00 for EC and −0.08 and −0.15 for NO_2_ for outdoors and indoors, respectively, all *p* > 0.30). Correlations between modeled BC and NO_2_ at home and measured EC and NO_2_ at school were weak (*r* = 0.27, *p <* 0.001, and *r* = 0.35, *p <* 0.001, respectively). Noise was moderately correlated with traffic pollutants (*r* = 0.46, *p* = 0.01, and *r* = 0.43, *p* = 0.01, for indoor EC and NO_2_, respectively).

**Table 3:** Description of the air pollutants at the 39 schools.

School Air Pollutant	Minimum	Percentile			Maximum
		25th	50th	75th	
EC outdoor	0.58	1.03	1.32	1.73	3.89
EC indoor	0.44	0.86	1.26	1.78	3.47
NO_2_ outdoor	25.9	35.1	48.5	57.4	84.5
NO_2_ indoor	11.5	20.5	29.8	38.6	65.6
UFP outdoor	11,939	16,27	22,157	28,257	51,146
UFP indoor	8,034	11,096	14,407	19,968	26,665

Units are micrograms per cubic meter (EC and NO_2_) or number per cubic centimeter (UFP). Median NO_2_ at the reference urban background station = 41 μg/m^3^.

**Table 4:** Correlation coefficients (Spearman) between air pollutants by season.

	EC (out)	NO_2_ (out)	UFP (out)	EC (in)	NO_2_ (in)	UPF (in)
**EC (out)**	0.58***	0.73***	0.62***	0.82***	0.53**	0.49**
**NO**_**2**_ **(out)**	0.63***	0.49**	0.51**	0.61***	0.71***	0.34
**UPF (out)**	0.61***	0.61***	0.72***	0.49**	0.30	0.57***
**EC (in)**	0.86***	0.69***	0.63***	0.73***	0.66***	0.61***
**NO**_**2**_ **(in)**	0.45**	0.70***	0.43*	0.58***	0.64***	0.39*
**UFP (in)**	0.41*	0.42*	0.65***	0.62***	0.38*	0.40*

Below diagonal, cold season (November–March); above diagonal, warm season (April–October). Correlations between the two seasons in the diagonal.

*p < 0.05

**p < 0.01

***p < 0.001.

out, outdoors (courtyard); in, indoors (classroom).

High- and low-exposed schools were comparable in terms of socioeconomic status, although low exposed schools had a higher socioeconomic vulnerability index (i.e., more deprived), were more likely to be public, had higher greenness, and were farther from the busy roads than high-exposed schools (Table 5). Quality of education was identical. However, children attending low-exposed schools had slightly better maternal education; had less behavioral problems, obesity, and foreign origin; had more siblings and residential greenness; and lived farther from the school and commuted less by walking than children from high-polluted schools (Table 5).

**Table 5:** Population and school characteristics by school traffic (from original design).

Characteristic	Low Traffic	High Traffic	*p*-Value^‡^
**Schools**			
Number	20	19	
School socioeconomic vulnerability index	0.52 (0.24)	0.41 (0.16)	0.055
School greenness (NDVI)	0.31 (0.10)	0.15 (0.03)	<0.001
Type of school, public	55%	42%	0.421
Educational quality (PISA 2012)	3.9 (1.3)	3.9 (1.8)	0.790
Noise level in classroom (decibels)	37.2 (4.9)	40.1 (5.0)	0.068
Distance to busy road (meters)	369 (357)	118 (178)	<0.001
EC outdoor (μg/m^3^)	1.13 (0.39)	1.82 (0.70)	<0.001
NO_2_ outdoor (μg/m^3^)	40.5 (9.6)	56.1 (11.5)	<0.001
UFP outdoor (number/cm^3^)	18,043 (5,702)	28,745 (8,326)	0.001
**Children**			
Number	1,355	1,360	
Girls	49%	51%	0.318
Foreign origin (non-Spanish)	11%	19%	<0.001
Maternal education, university	62%	55%	<0.001
Paternal education, university	58%	48%	<0.001
Maternal occupation, unemployed	17%	19%	0.036
Paternal occupation, unemployed	8%	12%	<0.001
Marital status, married	86%	84%	0.053
Home socioeconomic vulnerability index	0.43 (0.22)	0.47 (0.19)	<0.001
Home greenness (NDVI)	0.022 (0.09)	0.017 (0.005)	<0.001
Commuting to school, walking	33%	73%	<0.001
Distance from home to school (meters)	2,430 (2,359)	1,028 (1,577)	<0.001
Behavioral problems (SDQ)	7.9 (5.0)	8.9 (5.4)	<0.001
Overweight/obese	25%	30%	0.002
Computer games weekend, ≥1 h	69%	72%	0.081
Siblings, yes	83%	75%	<0.001
Adopted child	4%	4%	0.793
Secondhand smoke at home	12%	14%	0.069
Smoking during pregnancy	10%	10%	0.785
Gestational age < 37 wk	8%	7%	0.497
Birth weight < 2,500 g	9%	10%	0.994
Breastfeeding, no	18%	18%	0.272

Data are number, percent, or mean (standard deviation).

^‡^Kruskal-Wallis and Chi-square tests.

PISA, Programme for International Student Assessment.

### Association of High Versus Low Traffic Exposure with Cognitive Development

**Fig 2:**
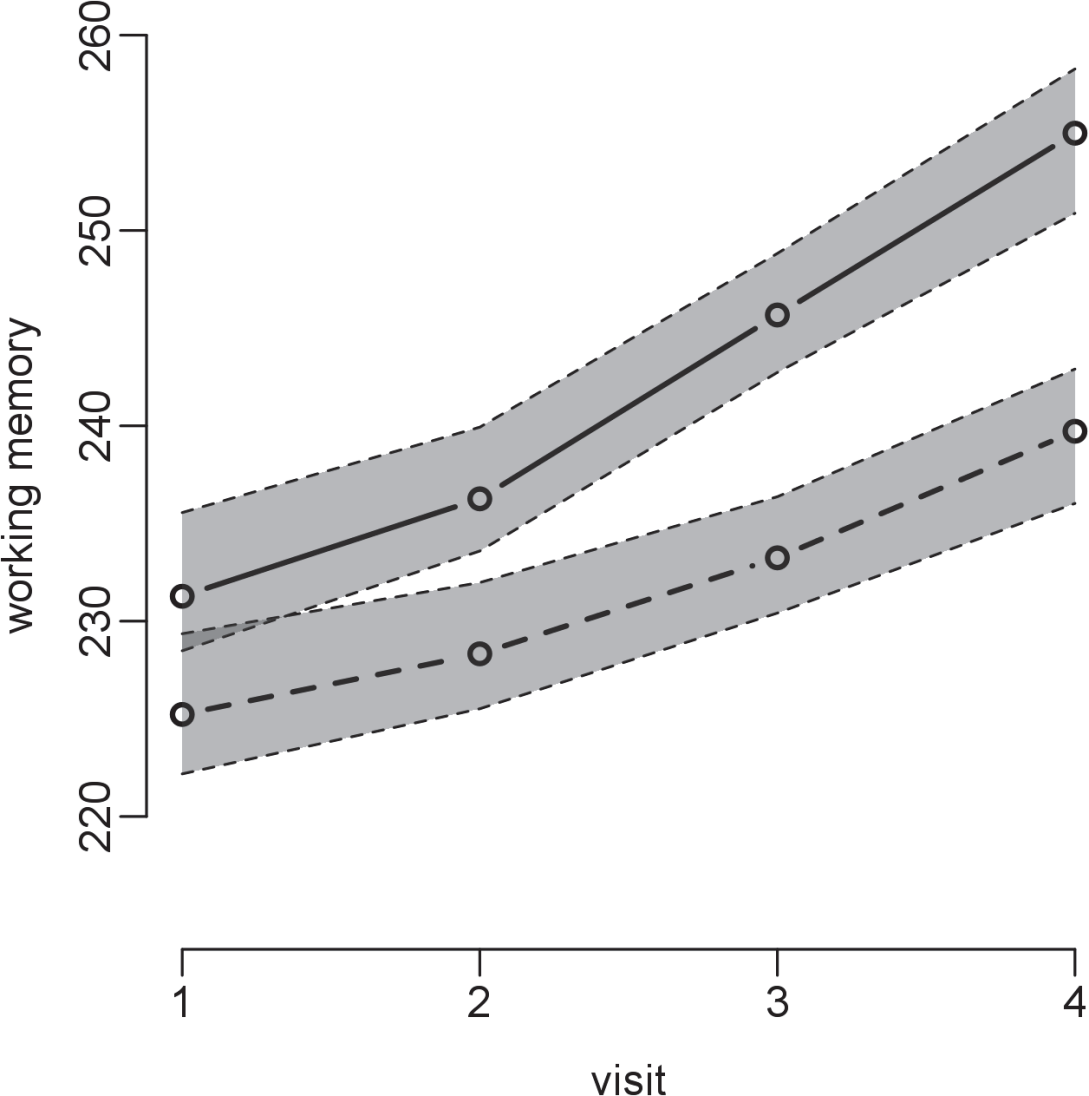
Working memory development by high- or low-traffic-air-pollution school. Dashed line = high traffic air pollution; continuous line = low traffic air pollution; gray shading indicates 95% CIs. Adjusted for age, sex, maternal education, residential neighborhood socioeconomic status, and air pollution exposure at home; school and individual as nested random effects in 2,715 children and 10,112 tests from 39 schools.

**Fig 3:**
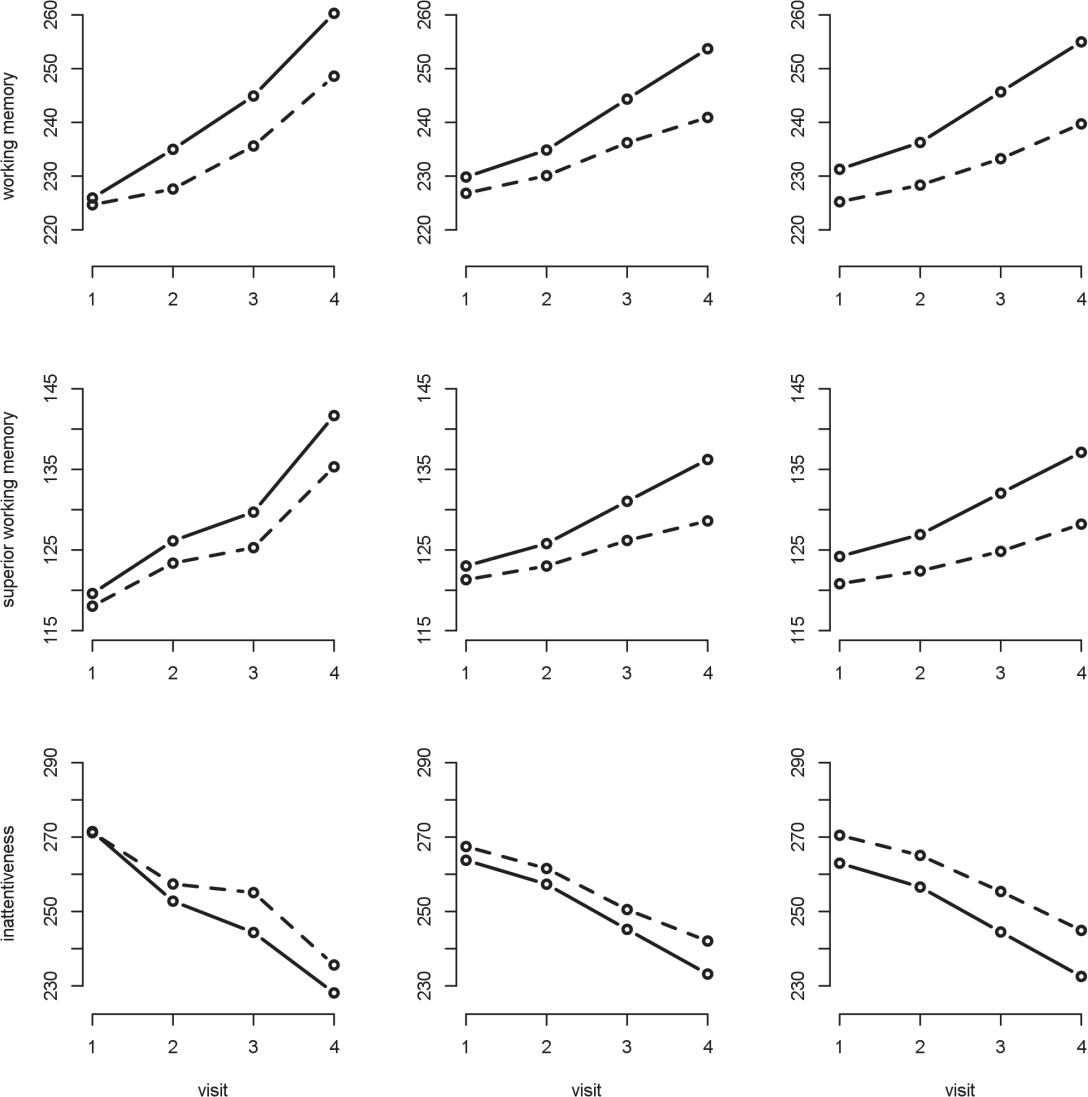
Crude and adjusted cognitive development by high- or low-air-pollution school. Dashed line = high air pollution; continuous line = low air pollution. The first column depicts the crude values, the second the crude trajectories from a model that included individual and school as random effects, and the third a model adjusted for age, sex, maternal education, residential neighborhood socioeconomic status, and air pollution exposure at home; school and individual as nested random effects in 2,715 children and 10,112 tests from 39 schools.

The difference in 12-mo change in working memory between the low- and high-exposed schools was statistically significant (Table 6). At baseline the difference in working memory between low- and high-exposure schools was 5.3 points, while after 1 y this difference had increased to 9.9 points (Table 6), which represents a 4.1% (95% CI 1.5%–6.8%, *p* = 0.0024) increase in the difference in working memory. Thus, children from high-air-polluted schools had lower improvement in cognitive development compared to children from the paired low-polluted schools (e.g., 7.4%, 95% CI 5.6%–8.8%, versus 11.5%, 95% CI 8.9%–12.5%, 12-mo increase in working memory) (Fig. 2). Similar effects were found for the other cognitive parameters (Fig. 3).

**Table 6:** Difference in cognitive development, at baseline and 12-mo change, by school air pollution exposure (high/low group or interquartile range increase) in 2,715 children and 10,112 tests from 39 schools.

Cognitive Outcome	High/Low Traffic	Outdoor (Courtyard)			Indoor (Classroom)		
		EC	NO_2_	UFP	EC	NO_2_	UFP
**Units per interquartile range**	—	0.7 μ g/m^3^	23.3 μg/m^3^	6,110 counts	0.92 μg/m^3^	18.1 μg/m^3^	8,872 counts
**Working memory (two-back numbers, *d′*)**							
Baseline	−5.3 (−16, 5.1)	−5.8 (−12, 0.56)	−7.5 (−16, 0.99)	−6.4 (−14, 1.5)	−3.0 (−11, 4.8)	−6.1 (−14, 1.9)	−1.3 (−13, 9.9)
12-mo change	−9.9 (−16, −3.5)*	−4.1 (−8.0, −0.2)*	−6.6 (−12, −1.2)*	−4.9 (−10, 0.22)	−6.2 (−11, −2.0)*	−5.6 (−11, −0.44)*	−7.9 (−15, −1.3)*
**Superior working memory (three-back numbers, *d′*)**							
Baseline	−1.4 (−10, 7.1)	0.25 (−5.2, 5.7)	1.5 (−5.8, 8.8)	−0.95 (−7.4, 5.6)	1.4 (−5.0, 7.9)	1.3 (−5.4, 8.0)	−0.078 (−9.1, 8.9)
12-mo change	−5.8 (−11, −0.74)*	−4.4 (−7.6, −1.3)*	−6.7 (−11, −2.3)*	−5 (−9.1, −0.96)*	−5.8 (−9.2, −2.4)*	−5.1 (−9.2, −0.91)*	−6.0 (−11, −0.75)*
**Inattentiveness (HRT-SE, milliseconds)**							
Baseline	5.2 (−6.2, 17)	1 (−6.3, 8.4)	4.8 (−5.0, 14)	4.5 (−4.0, 13)	6.8 (−1.7, 15)	7.0 (−1.8, 16)	6.2 (−5.8, 18)
12-mo change	5.2 (0.68, 9.7)*	3.8 (1.0, 6.6)*	3.8 (−0.10, 7.6)	3.9 (0.31, 7.6)*	3.8 (0.79, 6.8)*	2.6 (−1.0, 6.3)	4.6 (−0.13, 9.2)

Difference (95% CI) in the 12-mo change adjusted for age, sex, maternal education, residential neighborhood socioeconomic status, and air pollution exposure at home; school and individual as nested random effects.

*p < 0.05.

### Association of Direct Measurements of Traffic Air Pollution with Cognitive Development

**Fig 4:**
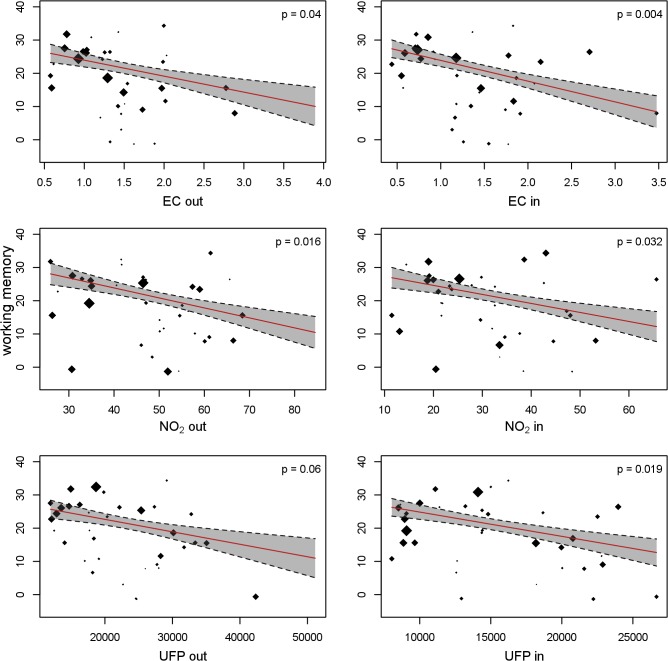
Working memory development and long-term exposure to traffic-related air pollutants. Each dot depicts a school, with size proportional to the number of children. The cognitive development per school was estimated in a model with school and individual as random effects. The slope of the red line depicts the change in cognitive development as a function of the air pollutants, adjusted for age, sex, maternal education, residential neighborhood socioeconomic status, and air pollution exposure at home; school and individual as nested random effects in 2,715 children and 10,112 tests from 39 schools. Gray shading indicates 95% CIs. out, outdoors (courtyard); in, indoors (classroom).

**Fig 5:**
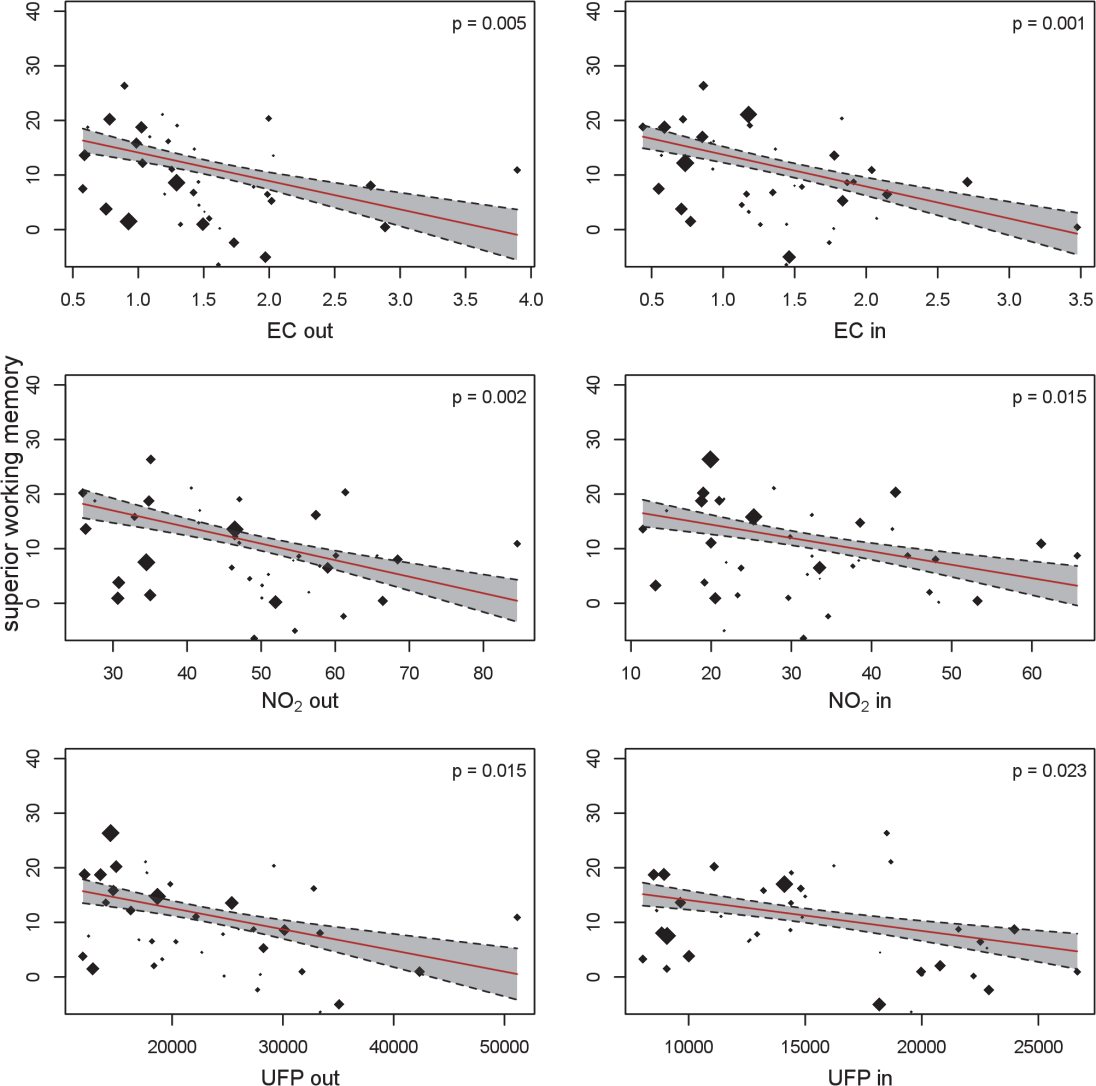
Superior working memory and long-term exposure to traffic-related air pollutants. Each dot depicts a school, with size proportional to the number of children. The cognitive development per school was estimated in a model with school and individual as random effects. The slope of the red line depicts the change in cognitive development as a function of the air pollutants, adjusted for age, sex, maternal education, residential neighborhood socioeconomic status, and air pollution exposure at home; school and individual as nested random effects in 2,715 children and 10,112 tests from 39 schools. Gray shading indicates 95% CI. out, outdoors (courtyard); in, indoors (classroom).

**Fig 6:**
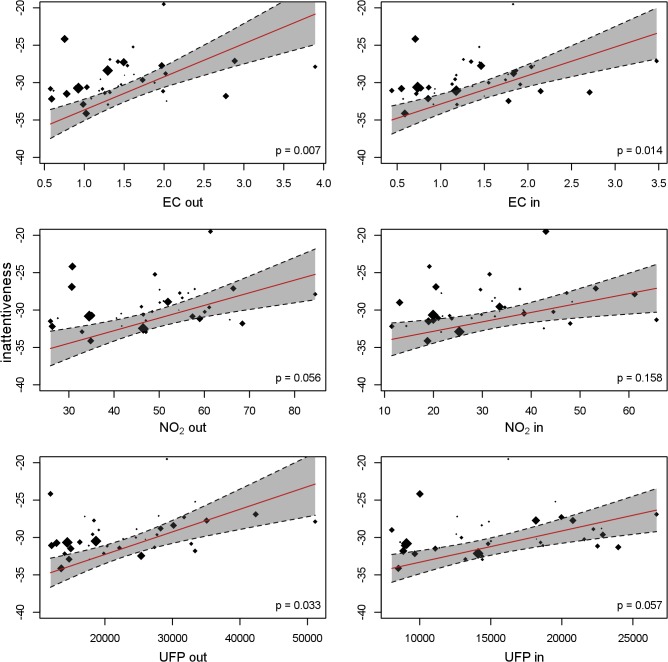
Inattentiveness development and long-term exposure to traffic-related air pollutants. Each dot depicts a school, with size proportional to the number of children. The cognitive development per school was estimated in a model with school and individual as random effects. The slope of the red line depicts the change in cognitive development as a function of the air pollutants, adjusted for age, sex, maternal education, residential neighborhood socioeconomic status, and air pollution exposure at home; school and individual as nested random effects in 2,715 children and 10,112 tests from 39 schools. Gray shading indicates 95% CI. out, outdoors (courtyard); in, indoors (classroom).

Table 6 gives the adjusted air pollution coefficients at baseline and per 12-mo change for all the cognitive parameters. Children attending schools with higher levels of EC, NO_2_, and UFP both in the courtyard and in the classroom had worse cognitive parameters at baseline than children attending schools with lower air pollution. All the coefficients were negative for working memory and positive for inattentiveness, indicating impairment, though the differences were not statistically significant. The growth in cognitive parameters during the 1-y follow-up was also reduced in the schools exposed to higher air pollution levels, which in consequence amplified the differences between schools at the end of follow-up. The detrimental association of air pollution with change in the cognitive parameters was observed for all the outcomes and pollutants, being statistically significant for almost all of them. Thus, for example, after 1 y of follow-up, the difference in working memory for a change from the first to the fourth quartile of indoor EC had increased by 6.2 (95% CI 2.0–11.0) points (*p* = 0.004) (13.0% [95% CI 4.2%–23.1%] of the total growth). When the stimulus was words instead of numbers, the results were very similar for superior working memory (Table 7). Fig. 4 shows the change in working memory in 1 y as a function of both outdoor and indoor pollutant levels. The points in the figure represent the crude estimates of change in cognitive parameters for each school along with the school air pollution levels, while the line represents the regression line obtained from the final adjusted model. Fig. 4 illustrates the negative relationship between change in cognitive function and air pollution levels, and depicts a good fit between the crude values and the adjusted slope. Similar findings were seen for the other cognitive parameters (Figs. 5 and 6).

**Table 7:** Difference in cognitive development for tests using words, at baseline and 12-mo change, by school air pollution exposure (high/low group or interquartile range increase) in 2,715 children and 10,112 tests from 39 schools.

Cognitive Outcome	High/Low Traffic	Outdoor (Courtyard)			Indoor (Classroom)		
		EC	NO_2_	UFP	EC	NO_2_	UFP
Working memory (two-back words, *d′*)							
Baseline	−8.4 (−19, 1.9)	−3.7 (−10, 2.9)	−3.3 (−12, 5.6)	−5.0 (−13, 3.0)	−4.1 (−12, 3.5)	−4.3 (−12, 3.8)	−4.3 (−15, 6.4)
12-mo change	−4.7 (−11, 1.7)	−1.7 (−5.6, 2.3)	−3.4 (−8.9, 2.1)	−3.1 (−8.2, 2.0)	−2.3 (−6.6, 2.0)	0.60 (−4.6, 5.8)	−5.4 (−12, 1.2)
**Superior working memory (three-back words, *d′*)**							
Baseline	−1.8 (−8.5, 4.9)	0.25 (−4.0, 4.5)	0.96 (−4.8, 6.7)	−0.67 (−5.9, 4.6)	0.88 (−4.0, 5.7)	0.096 (−5.2, 5.4)	0.40 (−6.7, 7.5)
12-mo Change	−5.9 (−11, −0.89)*	−4.9 (−8.0, −1.8)*	−6.8 (−11, −2.5)*	−5.7 (−9.7, −1.7)*	−5.4 (−8.7, −2.0)*	−3.9 (−8.0, 0.14)	−3.9 (−9.1, 1.3)

Difference (95% CI) in the yearly change adjusted for age, sex, maternal education, residential neighborhood socioeconomic status, and air pollution exposure at home; school and individual as nested random effects.

*p < 0.05

### Sensitivity Analyses

The crude and the adjusted models with high- versus low-air-pollution schools and with the direct measures of air pollutants gave similar results (Fig. 3; S1 Table). Further adjustment for the individual socioeconomic factors included in Table 5, ADHD or behavioral symptoms, residential greenness, and school noise and greenness did not materially change associations between high/low air pollution; EC, NO_2_, and UFP; and 12-mo change in cognitive parameters. Similarly, results remained unchanged after adjusting for high-/low-air-pollution area, commuting, smoking at home (S2 Table), educational quality, and participation rate per school.

In stratified analysis, associations of cognitive parameters with EC (Table 8), NO_2_, and UFP were similar in high-air-pollution schools and low-air-pollution schools, as well as according to neighborhood socioeconomic status and obesity. In contrast, detrimental associations were stronger in general in boys than in girls, in children from more highly educated mothers, in children from private schools, and in children with ADHD symptoms, though differences were not significant (*p* for interaction > 0.1 in the mixed effects linear models), and the detrimental associations occurred in all the groups. Given that development was significantly lower in grade 4 for all tasks, we repeated the analyses stratifying by grade, and the results were homogeneous. Moreover, in order to control for the “summer learning loss” phenomenon occurring between the two academic years, we excluded tests done in the second academic year that did not result in a notable change in our observed associations. Furthermore, we excluded the first exam, to prevent a potential practice effect, and the association, if anything, became stronger for working memory and superior working memory (S3 Table). Finally, sequential exclusion of school pairs one by one from the models did not change the results, suggesting that exceptional influential cases were not affecting the results.

**Table 8:** Stratified analyses of adjusted 12-mo change in cognitive development by school air pollution exposure (high/low group or interquartile range increase) in 2,715 children and 10,112 tests from 39 schools.

Cognitive Outcome	By Sex		By Maternal Education		By ADHD		By High/Low Air Pollution		By Type of School	
	Boys (*n* = 1,357)	Girls (*n* = 1,358)	High (*n* = 1,590)	Low–Middle (*n* = 1,125)	No (*n* = 2,409)	Yes (*n* = 275)	High (*n* = 1,358)	Low (*n* = 1,357)	Public (*n* = 931)	Private (*n* = 1,784)
**Working memory (two-back numbers, *d′*)**										
High/low	−13 (−23, −4.2)*	−6.1 (−15, 2.6)	−15 (−23, −6.4)*	−3.2 (−13, 6.7)	−7.7 (−14, −0.97)*	−26 (−45, −6.7)*	−	−	−0.15 (−12, 11)	−14 (−22, −6.4)*
EC outdoor	−6.4 (−12, −0.75)*	−1.3 (−6.7, 4.0)	−10 (−15, −5.1)*	4 (−2.2, 10)	−1.9 (−6.0, 2.3)	−17 (−29, −5.6)*	1.2 (−4.6, 6.9)	−6.9 (−16, 2.4)	3.9 (−3.0, 11)	−8.0 (−13, −3.1)*
EC indoor	−8.9 (−15, −2.8)*	−3.2 (−9.1, 2.8)	−10 (−16, −4.7)*	−0.64 (−7.5, 6.2)	−3.5 (−8.0, 1.1)	−22 (−35, −8.5)*	−2.7 (−8.8, 3.5)	−6.6 (−18, 5.0)	−0.53 (−11, 10)	−7.1 (−12, −2.3)*
**Superior working memory (three-back numbers, *d′*)**										
High/low	−10 (−18, −3.0)*	−1.9 (−8.8, 5.0)	−7.5 (−14, −0.74)*	−3.7 (−11, 4.0)	−5.2 (−11, 0.14)	−12 (−26, 3.0)	−	−	−2.1 (−11, 7.1)	−7.3 (−13, −1.2)*
EC outdoor	−9.6 (−14, −5.1)*	1.2 (−3.1, 5.5)	−6.7 (−11, −2.6)*	−1.2 (−6.0, 3.6)	−3.3 (−6.7, 0.03)	−11 (−19, −1.8)*	−3.1 (−7.8, 1.5)	−4.8 (−12, 2.5)	−1.8 (−7.3, 3.7)	−5.5 (−9.4, −1.6)*
EC indoor	−10 (−15, −5.4)*	−0.85 (−5.6, 3.9)	−8.9 (−13, −4.5)*	−1.4 (−6.7, 3.9)	−4.7 (−8.4, −1.1)*	−11 (−20, −0.95)*	−5.7 (−11, −0.71)*	−4.2 (−13, 4.9)	−4.9 (−13, 3.4)	−5.7 (−9.6, −1.9)*
**Inattentiveness (HRT-SE, milliseconds)**										
High/low	8.1 (1.8, 15)*	1.4 (−4.9, 7.8)	9.0 (3.1, 15)*	−0.93 (−8.2, 6.3)	5.5 (0.69, 10)*	3.6 (−11, 18)	−	−	1.1 (−7.1, 9.2)	7.9 (2.4, 13)*
EC outdoor	5.8 (1.9, 9.6)*	1.8 (−2.2, 5.7)	5.2 (1.7, 8.7)*	1.4 (−3.0, 5.9)	2.3 (−0.63, 5.2)	13 (4.9, 22)*	4.7 (0.72, 8.8)*	−2 (−8.6, 4.5)	3.6 (−1.1, 8.3)	4.5 (1.0, 8.0)*
EC indoor	5.2 (1.0, 9.4)*	2.0 (−2.3, 6.4)	4.6 (0.84, 8.4)*	1.9 (−3.1, 6.8)	1.9 (−1.3, 5.2)	16 (7.0, 26)*	3.9 (−0.47, 8.2)	−2 (−10, 6.2)	5.3 (−2.0, 13)	4.2 (0.74, 7.6)*

Difference (95% CI) in the 12-mo change, adjusted for age, sex, maternal education, residential neighborhood socioeconomic status, and air pollution exposure at home; school and individual as nested random effects.

*p < 0.05.

## Discussion

This large study with repeated and objective measures demonstrated that cognitive development is reduced in children exposed to higher levels of traffic-related air pollutants at school. This association was consistent for working memory, superior working memory, and inattentiveness, and robust to several sensitivity analyses. The association was observed both when the exposure was treated as high/low traffic-related air pollution and when using specific pollutants including outdoor and indoor EC, NO_2_, and UFP, which are largely traffic-related [21,22]. Changes in the developmental trajectory could resemble those suggested for the adverse impact of urban air pollution on lung function development [29]. Mechanisms of air-pollution-induced neurotoxicity have been explored [30]. The findings provide strong support for air pollution being a developmental neurotoxicant and point towards the primary school age as a particularly vulnerable time window for executive function development.

A strength of this study is the longitudinal ascertainment of executive function trajectories that specifically develop during school age and the direct measures of air pollution. A concern, however, is potential residual confounding by socio-demographic characteristics, although in European cities, the relationship between proximity to traffic and economically disadvantaged areas is not always evident [31]. In the city of Barcelona, the highest air pollution was observed in the “Eixample,” a wealthy central area of the city where most of our schools with high traffic were selected [23]. We paired by design high- and low-traffic schools by socioeconomic characteristics and type of school, and although there was an inverse relation between school pollution and socioeconomic vulnerability index, such differences between schools after matching became small. In addition to the association of cognitive parameters observed with high- compared to low-exposed schools, we also observed a consistent association of cognitive parameters with specific pollutants whose relation with socio-demographics was weak and in some cases nonexistent. Furthermore, cognitive development was unrelated to social determinants in our study, in contrast to cognitive function at baseline. Besides, the associations remained in the stratified analyses (e.g., for type of school or high-/low-polluted area) and after additional adjustment (e.g., for commuting, educational quality, or smoking at home), contradicting a potential residual confounding explanation.

Other potential limitations are the potential misclassification error of the UFP exposures. Seasonalized measures of UFP showed the lowest correlation among the pollutants between the first and the second campaign and weaker associations with the cognitive parameters (e.g., −4.0 [95% CI −8.6 to 0.49] for indoor UFP and working memory) than non-seasonalized UFP, which is probably because of its large geographical and temporal instability due to constant and rapid secondary formation [22]. In contrast, EC and NO_2_ showed very similar associations with cognitive parameters using both seasonalized and non-seasonalized measures. Another potential limitation is non-response. A total of 182 out of the initial 2,897 children (6%) were excluded because of incomplete data on individual variables. When these children were included in the analysis in models that did not require the complete dataset (i.e., a model not adjusted for maternal education), results were identical. Another level of non-response refers to children (41%) from families that did not want to be part of the study, although they were invited. This non-response affects representativeness rather than internal validity, given that the participation rate per school was unrelated to the school social gradient and that adjustment for participation rate did not change the results. Based on the results from one school, participants had less neuropsychological problems than non-participants, which likely made them less susceptible to air pollution effects. Therefore, any effect observed in the present study would likely be a conservative estimate for extrapolation to the entire population. A third limitation relates to the lack of measurements in preceding periods. However, all children had been in their school for more than 6 mo before the beginning of the study, and when we limited the study to children with more than 2 y in the school (94% of the children), associations remained the same. We interpreted these associations as chronic effects (i.e., due to exposures longer than 6 mo) since it is unlikely that the geographical pattern of air pollution occurring during the study period had changed in the last 2 y. Finally, indoor assessment was limited to a single classroom. This is not a problem for the indoor assessment of pollutants such as EC, given the high correlation between outdoor and indoor levels and similar coefficients for the association with cognition between outdoor and indoor exposures. However, it could be a problem for school noise since the correlation between outdoor and indoor noise was strongly dependent on the street orientation of the classroom (ranging from 0.07 for classrooms facing away from the street to 0.70 for classrooms facing the street). However, residual confounding by noise was unlikely given the weak correlation between the pollutants and noise measured in the same classrooms, and the robustness of the coefficients for the different pollutants after adjusting for noise and for the interaction between noise and age.

This study addresses the role of traffic air pollution in schools on cognitive development. Previous studies on the effects of polluted air at schools were a study in two schools in Quanzhou (China) on attention disorders [10], two studies on aircraft noise that secondarily assessed the association between NO_2_ and cognitive function [32,33], and an ecological study in Michigan (US) on industrial pollution and school failure [34]. Other studies in children have evaluated the effect of maternal personal air pollution exposure or maternal/child exposure at home [35]. We found here an association between traffic-related air pollution exposure at school and cognitive development during primary school age, independent of residential air pollution and beyond the effects related to home exposures in early life found by previous studies. Total cumulative exposure in school, home, and commuting and the different time windows of exposure are not addressed here, but the continuous monitoring of BC and physical activity with personal samplers in 54 of our children showed that exposure at school was significantly higher than at home and did not change by commuting mode. This higher exposure level at school could be attributed to peaks of pollution occurring during school time, and higher inhaled dose during school time due to exercise and physical activity at schools. Besides, the fact that children at schools in the most polluted area traveled a shorter distance from home suggests a shorter commute, which could explain the lack of confounding after adjusting for commuting distance and mode. We could not disentangle the time frame of the exposures occurring under the long-term school exposure measured here. However, in the case of inattentiveness, in contrast to what was seen for working memory, the association at baseline was larger than at follow-up. Given that inattentiveness develops earlier than working memory [12], this finding could suggest that the adverse effect of air pollution could have preceded the study period, and that the lower improvement in scores may be associated with previous exposures, too.

Among the individual traffic-related pollutants, we found an adverse association between EC and child cognitive development. EC comes almost exclusively from diesel vehicles in Barcelona, with an ambient air mode of around 30–40 nm, in the UFP range [22]. EC and traffic-derived metals were an important fraction of indoor and outdoor quasi-ultrafine particles (PM_0.25_) in our study schools [36]. We observed a high penetration of EC into the classrooms (indoor/outdoor ratio 94%) and similar associations of indoor and outdoor EC with cognitive development. Although the indoor/outdoor ratio was weaker (70%) for UFP, we also found cognitive associations with UFP. These findings remained after adjustment for traffic noise at school, pointing towards UFP as a neurotoxic traffic component, which is coherent with the numerous and consistent findings in animal studies that UFP may cause disruption of the blood–brain barrier, microglial activation, and brain inflammation [14].

Evidence points towards chronic microglial stimulation and altered innate immune response and inflammation as the key neurotoxic mechanisms of UFP [14,29,37]. UFP has been shown to cause microglial inflammation following either brain UFP deposition or systemic inflammation originating in UFP-exposed organs such as the lungs [36]. Microglial stimulation affects neurons, and UFP has been shown to decrease neuronal glutamatergic function and disrupt synapses [38]. Similarly, airborne metals have been shown to alter dopamine function [39]. The underlying brain mechanisms are beyond the present study, but the observation of associations with executive functions, the lack of confounding by ADHD or behavior, and the association among children without ADHD suggests a general brain dysfunction.

Boys appeared more susceptible to air pollution, although both boys and girls showed an adverse association of school air pollution with cognitive development. Although results could be due to chance, in animals, males were more susceptible to airborne metals than females, which may be because of sex-specific altered dopamine function [39]. The possible higher vulnerability of children with ADHD could also indicate abnormalities related to dopamine [40]. Stratification by maternal education or type of school showed a larger association among students with the most educated mothers and those from private schools. This resembles what has been observed with other hazards for neurodevelopment such as genetic effects [41], presumably because fewer adverse factors play a role among students with educated mothers or in private schools, thus causing less interference with the factors under study.

The observed association between air pollution and cognitive development was strong. For example, an increase from the first to the fourth quartile in indoor EC resulted in a 13.0% reduction in the growth of working memory. In contrast, the association at baseline was smaller (1.9%). Part of this larger association during primary school may be a matter of bigger magnitude of exposure to traffic pollution in schools, but it could indicate that some executive functions are particularly vulnerable during primary school age, as has also been seen for lead [42]. The long-term effect probably occurs over the period of maximum development of these executive functions, resulting in a notable cumulative effect by the end of this period in preadolescence. The observed association was consistent across cognitive measurements, though it was more evident for superior working memory, which is a good predictor of learning achievement [19]. Impairment of high cognitive functions has severe consequences for school achievement [11]. Thus, reduced cognitive development in children attending the most polluted schools might result in a disadvantage in mental capital, which may have a long-lasting life course effect.

Overall, we have shown that children attending schools with higher levels of exposure to traffic-related air pollutants had a smaller growth in cognitive development over time, suggesting that traffic-related air pollution in schools negatively affects cognitive development. This may have consequences for learning, school achievement, and behavior. With regard to air pollution regulation, the present study shows that the developing brain may be vulnerable to certain traffic-related air pollutants.

## Supporting Information

S1 Table Crude difference (and 95% CI) in cognitive development at baseline and 12-mo change by school air pollution exposure (high versus low or interquartile range increase) in 2,715 children and 10,112 tests from 39 schools. (DOCX)

S2 Table Difference (and 95% CI) in cognitive development at baseline and 12-mo change by school air pollution exposure (high versus low or interquartile range increase) in 2,715 children and 10,112 tests from 39 schools, after further adjustment for high/low area, commuting, and smoking at home. (DOCX)

S3 Table Difference (and 95% CI) in cognitive development (12-mo change) by school air pollution exposure (high/low group or interquartile range increase) in 2,715 children and 10,112 tests, after excluding some child-visits. (DOCX)

S1 Text STROBE checklist. (DOCX)

## Abbreviations:

ADHD: Attention deficit hyperactivity disorder
ANT: attentional network test
BC: black carbon
EC: elemental carbon
HRT-SE: hit reaction time standard error
NDVI: Normalized Difference Vegetation Index
PM_2.5_: particulate matter < 2.5 μm
SDQ: Strengths and Difficulties Questionnaire
UFP: ultrafine particle number
