# Supplementary material for: Association between Traffic-Related Air Pollution in Schools and Cognitive Development in Primary School Children: A Prospective Cohort Study
Source: PLoS Med. 2015 Mar 3;12(3):e1001792. doi: 10.1371/journal.pmed.1001792 (PMC4348510; doi:10.1371/journal.pmed.1001792)
Supplement: S1 Table — (DOCX) [file pmed.1001792.s001.docx]

| **S1 Table:** Crude difference (and 95% confidence interva)l‡ in cognitive development at baseline and 12-month change, per school air pollution exposure (high-low group or interquartile range increase, IQR) in 2715 children and 10,112 tests from 39 schools. | | | | | | | |
| --- | --- | --- | --- | --- | --- | --- | --- |
|  | **High/Low Traffic** | **Outdoor (courtyard)** | | | **Indoor (classroom)** | | |
|  |  | **EC** | **NO_2_** | **UFP** | **EC** | **NO_2_** | **UFP** |
| **Working memory (WM)** (2-back Numbers, d’) |  |  |  |  |  |  |  |
| Baseline | -4.7 (-18, 8.6) | -8 (-16, 0.0055) | -9.5 (-20, 1.2) | -11 (-20, -2.2)* | -4.5 (-15, 5.6) | -4.9 (-15, 5.7) | -5.6 (-19, 8.3) |
| 12-month change | -11 (-18, -4.9)* | -3.2 (-7.1, 0.68) | -5.6 (-11, -0.17)* | -5.1 (-10, -0.13)* | -5.5 (-9.8, -1.1)* | -4.3 (-9.5, 0.86) | -7.7 (-14, -1.1)* |
| **Superior WM** (3-back Numbers, d’) |  |  |  |  |  |  |  |
| Baseline | -4.1 (-14, 6.3) | -3 (-9.7, 3.7) | -2.2 (-11, 6.7) | -7 (-14, 0.31) | -1.1 (-9.2, 7.1) | 0.82 (-7.7, 9.3) | -5.2 (-16, 5.6) |
| 12-month change | -6 (-11, -0.92)* | -3.5 (-6.6, -0.4)* | -5.1 (-9.4, -0.85)* | -4.2 (-8.2, -0.25)* | -5.2 (-8.6, -1.8)* | -3.9 (-8, 0.27) | -5.9 (-11, -0.66)* |
| **Inattentiveness** (HRT-SE, ms) |  |  |  |  |  |  |  |
| Baseline | 4.3 (-10, 19) | 2.9 (-6.4, 12) | 6.5 (-5.6, 19) | 9.6 (-0.47, 20) | 7.2 (-3.7, 18) | 4.9 (-6.6, 16) | 9.4 (-5.3, 24) |
| 12-month change | 6.2 (1.7, 11)* | 3.6 (0.87, 6.3)* | 3.4 (-0.37, 7.3) | 3.9 (0.36, 7.5)* | 3.5 (0.48, 6.6)* | 2.3 (-1.4, 5.9) | 5 (0.32, 9.6)* |
| * p<0.05 | | | | | | | |
| ^‡^ Difference in the yearly change; school and subject as nested random effects. | | | | | | | |
| EC: Elemetal Carbon; NO_2_: Nitrogen Dioxide; UFP: number of Ultrafine Particles; HRT: Hit Reaction Time; SE: Standar error; d': detectability | | | | | | | |
