# Supplementary material for: Association between Traffic-Related Air Pollution in Schools and Cognitive Development in Primary School Children: A Prospective Cohort Study
Source: PLoS Med. 2015 Mar 3;12(3):e1001792. doi: 10.1371/journal.pmed.1001792 (PMC4348510; doi:10.1371/journal.pmed.1001792)
Supplement: S2 Table — (DOCX) [file pmed.1001792.s002.docx]

| **S2 Table:** Difference (and 95% confidence interval)^‡^ in cognitive development (12-month change), per school air pollution exposure (high-low group or interquartile range increase, IQR) in in 2,715 children and 10,112 tests and after further adjustment for high-low area, commuting and smoking at home. | | | | | |
| --- | --- | --- | --- | --- | --- |
|  | **Original model^‡^** | | **Adjusted for** | | |
|  | All (n= 2,715) | Restricted^¶^ (n=2,376) | High/Low (n= 2,715) | Commutting^¶^  (n=2,376) | Smoking^¶^  (n=2,376) |
| **Working memory** (2-back Numbers, d’) |  |  |  |  |  |
| High/Low | -9.9 (-16, -3.5) * | -11 (-18, -4) * |  | -11 (-18, -4.2) * | -11 (-18, -4.1) * |
| EC-outdoor | -6.2 (-11, -2) * | -6.6 (-11, -2.2) * | -6.5 (-11, -2.1) * | -6.6 (-11, -2.2) * | -6.7 (-11, -2.3) * |
| EC-indoor | -5.6 (-11, -0.44) * | -6.7 (-12, -1) * | -5.9 (-11, -0.55) * | -6.8 (-12, -1.1) * | -6.8 (-12, -1.1) * |
| NO2-outdoor | -7.9 (-15, -1.3) * | -8 (-15, -0.85) * | -8.2 (-15, -1.3) * | -8.1 (-15, -0.99) * | -8.2 (-15, -1) * |
| NO2-indoor | -4.1 (-8.1, -0.2) * | -4.7 (-8.8, -0.63) * | -4.2 (-8.2, -0.2) * | -4.7 (-8.8, -0.66) * | -4.7 (-8.8, -0.65) * |
| UPF-outdoor | -6.6 (-12, -1.2) * | -7.8 (-14, -2) * | -6.9 (-12, -1.3) * | -7.9 (-14, -2.1) * | -7.8 (-14, -2) * |
| UPF-indoor | -4.9 (-10, 0.22) | -5.2 (-11, 0.21) | -5.1 (-10, 0.18) | -5.3 (-11, 0.15) | -5.2 (-11, 0.21) |
| **Superior WM** (3-back Numbers, d’) |  |  |  |  |  |
| High/Low | -5.8 (-11, -0.74) * | -7.8 (-13, -2.4) * |  | -8.1 (-13, -2.6) * | -7.8 (-13, -2.4) * |
| EC-outdoor | -5.8 (-9.2, -2.4) * | -6.2 (-9.7, -2.7) * | -6.2 (-9.6, -2.7) * | -6.3 (-9.8, -2.7) * | -6.2 (-9.7, -2.6) * |
| EC-indoor | -5.1 (-9.2, -0.91) * | -5.4 (-9.9, -0.83) * | -5.4 (-9.6, -1.1) * | -5.5 (-10, -0.94) * | -5.4 (-9.9, -0.81) * |
| NO2-outdoor | -6 (-11, -0.75) * | -6.1 (-12, -0.4) * | -6.1 (-12, -0.7) * | -6.3 (-12, -0.6) * | -6.1 (-12, -0.36) * |
| NO2-indoor | -4.4 (-7.6, -1.3) * | -5.3 (-8.6, -2) * | -4.5 (-7.7, -1.4) * | -5.3 (-8.6, -2.1) * | -5.3 (-8.6, -2) * |
| UPF-outdoor | -6.7 (-11, -2.3) * | -7.4 (-12, -2.8) * | -7.3 (-12, -2.9) * | -7.5 (-12, -2.9) * | -7.4 (-12, -2.8) * |
| UPF-indoor | -5 (-9.1, -0.96) * | -6 (-10, -1.6) * | -4.6 (-8.8, -0.39) * | -6 (-10, -1.7) * | -5.9 (-10, -1.6) * |
| **Inattentiveness** (HRT-SE, ms) |  |  |  |  |  |
| High/Low | 5.2 (0.68, 9.7) * | 7.1 (2.2, 12) * |  | 7.3 (2.5, 12) * | 7 (2.2, 12) * |
| EC-outdoor | 3.8 (0.79, 6.8) * | 4.4 (1.3, 7.5) * | 5 (0.93, 7) * | 4.4 (1.3, 7.6) * | 4.4 (1.2, 7.5) * |
| EC-indoor | 2.6 (-1, 6.3) | 4 (0.04, 8) * | 2.7 (-1, 6.4) | 4.1 (0.12, 8.1) * | 4 (0.033, 8) * |
| NO2-outdoor | 4.6 (-0.13, 9.2) | 4.8 (-0.27, 9.8) | 4.9 (0.11, 9.6) * | 4.9 (-0.09, 10) | 4.7 (-0.29, 9.8) |
| NO2-indoor | 3.8 (1, 6.6) * | 4.4 (1.5, 7.3) * | 3.7 (0.96, 6.5) * | 4.4 (1.5, 7.3) * | 4.4 (1.5, 7.3) * |
| UPF-outdoor | 3.8 (-0.1, 7.6) | 5.1 (0.99, 9.1) * | 5 (0.066, 7.8) * | 5.1 (1, 9.2) * | 5.1 (0.99, 9.1) * |
| UPF-indoor | 3.9 (0.31, 7.6) * | 4.5 (0.65, 8.4) * | 3.6 (-0.069, 7.3) | 4.6 (0.73, 8.4) * | 4.5 (0.65, 8.4) * |
| * p<0.05 | | | | | |
| ^‡^ Difference in the 12-months change adjusted for age, sex, maternal education, residential neighbourhood socio-economic status and air pollution exposure at home; school and subject as nested random effects. | | | | | |
| ¶ Children with no data on commutting and smoking are excluded, in order to be compared with models adjusted for commutting (distance home school and % walking to school) or smoking at home. | | | | | |
| EC: Elemetal Carbon; NO2: Nitrogen Dioxide; UFP: number of Ultrafine Particles; HRT: Hit Reaction Time; SE: Standar error; d': detectability; | | | | | |
