# Supplementary material for: Association between Traffic-Related Air Pollution in Schools and Cognitive Development in Primary School Children: A Prospective Cohort Study
Source: PLoS Med. 2015 Mar 3;12(3):e1001792. doi: 10.1371/journal.pmed.1001792 (PMC4348510; doi:10.1371/journal.pmed.1001792)
Supplement: S3 Table — (DOCX) [file pmed.1001792.s003.docx]

| **S3 Table:** Difference (and 95% confidence interval)^‡^ in cognitive development (12-month change), per school air pollution exposure (high-low group or interquartile range increase, IQR) in in 2,715 children and 10,112 tests and after excluding some tests. | | | | |
| --- | --- | --- | --- | --- |
|  | **All visits**  (n= 10,112 tests) | **Exclusion of tests done** | | |
|  |  | In September (n=10,016) | During the 2nd academic year (n= 5,893) | In visit 1 (n= 8,724) |
| **Working memory** (2-back Numbers, d’) |  |  |  |  |
| High/Low | -9.9 (-16, -3.5) * | -9.3 (-16, -2.9) * | -8.2 (-17, 0.49) | -9.9 (-17, -2.6) * |
| EC-outdoor | -6.2 (-11, -2) * | -6.1 (-10, -1.8) * | -6 (-12, -0.13) * | -7.3 (-12, -2.4) * |
| EC-indoor | -5.6 (-11, -0.44) * | -5 (-10, 0.26) | -4.4 (-12, 3.1) | -6.2 (-12, -0.19) * |
| NO_2_-outdoor | -7.9 (-15, -1.3) * | -7.8 (-15, -1.1) * | -4.1 (-13, 5) | -11 (-19, -3.4) * |
| NO_2_-indoor | -4.1 (-8.1, -0.2) * | -3.9 (-7.8, 0.087) | -4.6 (-9.1, -0.11) * | -5 (-9.5, -0.45) * |
| UPF-outdoor | -6.6 (-12, -1.2) * | -6.2 (-12, -0.73) * | -7.1 (-14, 0.3) | -8 (-14, -1.7) * |
| UPF-indoor | -4.9 (-10, 0.22) | -4.5 (-9.7, 0.58) | -5.9 (-13, 1) | -6.5 (-12, -0.67) * |
| **Superior WM** (3-back Numbers, d’) |  |  |  |  |
| High/Low | -5.8 (-11, -0.74) * | -5.6 (-11, -0.44) * | -9.5 (-16, -2.7) * | -5.7 (-12, 0.12) |
| EC-outdoor | -5.8 (-9.2, -2.4) * | -5.9 (-9.4, -2.5) * | -7.3 (-12, -2.7) * | -6.2 (-10, -2.3) * |
| EC-indoor | -5.1 (-9.2, -0.91) * | -5.2 (-9.4, -0.99) * | -3.3 (-9.2, 2.6) | -6.6 (-11, -1.9) * |
| NO_2_-outdoor | -6 (-11, -0.75) * | -6.1 (-12, -0.78) * | -9 (-16, -1.8) * | -5.7 (-12, 0.39) |
| NO_2_-indoor | -4.4 (-7.6, -1.3) * | -4.6 (-7.8, -1.5) * | -3.6 (-7.1, -0.055) * | -6.2 (-9.8, -2.6) * |
| UPF-outdoor | -6.7 (-11, -2.3) * | -6.7 (-11, -2.3) * | -5.2 (-11, 0.55) | -8 (-13, -3) * |
| UPF-indoor | -5 (-9.1, -0.96) * | -5.1 (-9.2, -1) * | -4.7 (-10, 0.79) | -7.1 (-12, -2.4) * |
| **Inattentiveness** (HRT-SE, ms) |  |  |  |  |
| High/Low | 5.2 (0.68, 9.7) * | 5.7 (1.1, 10) * | 3.1 (-3.4, 9.6) | 2.2 (-3.1, 7.5) |
| EC-outdoor | 3.8 (0.79, 6.8) * | 3.9 (0.87, 6.9) * | 5.2 (0.81, 9.5) * | 3 (-0.5, 6.6) |
| EC-indoor | 2.6 (-1, 6.3) | 3.1 (-0.6, 6.8) | 4.8 (-0.88, 10) | 2.4 (-1.9, 6.7) |
| NO_2_-outdoor | 4.6 (-0.13, 9.2) | 5.1 (0.37, 9.8) * | 6.5 (-0.34, 13) | 2.7 (-2.8, 8.1) |
| NO_2_-indoor | 3.8 (1, 6.6) * | 3.8 (1.1, 6.6) * | 4.4 (1, 7.8) * | 3.5 (0.3, 6.8) * |
| UPF-outdoor | 3.8 (-0.1, 7.6) | 3.7 (-0.22, 7.5) | 5.8 (0.28, 11) * | 3.6 (-0.92, 8) |
| UPF-indoor | 3.9 (0.31, 7.6) * | 4.1 (0.47, 7.7) * | 6.5 (1.2, 12) * | 2.9 (-1.3, 7.1) |
| * p<0.05 | | | | |
| ^‡^ Difference in the 12-months change adjusted for age, sex, maternal education, residential neighbourhood socio-economic status and air pollution exposure at home; school and subject as nested random effects. | | | | |
| EC: Elemetal Carbon; NO2: Nitrogen Dioxide; UFP: number of Ultrafine Particles; HRT: Hit Reaction Time; SE: Standar error; d': detectability; | | | | |
| "n" refers to the number of tests included in the model after the exclusion | | | | |
